# Core values of employed general practitioners in Germany – a qualitative study

**DOI:** 10.1186/s12875-023-02255-7

**Published:** 2024-01-06

**Authors:** Leonie Horn, Charlotte Ullrich, Leonie Boelter, Michel Wensing, Frank Peters-Klimm, Sandra Stengel

**Affiliations:** Department of general practice and health services research, University Hospital Heidelberg, Heidelberg University, Heidelberg, Germany

**Keywords:** Employment, Core values, Definition of general practice, Family medicine

## Abstract

**Background:**

“Core values” help to guide practice of health care delivery. The core values of general practice are described in the European definition of general practice by WONCA, e.g. a holistic, comprehensive and continuous care. They may be associated with the idea that the general practitioner is the owner of the practice rather than an employee.

**Objectives:**

The objective was to examine the core values of employed GPs in their professional setting and their practical manifestation.

**Methods:**

From April to May 2021, we conducted 17 semi-structured telephone-interviews with employed GPs in two districts in Baden-Wuerttemberg, Germany. The data were analysed using qualitative content analysis.

**Results:**

We identified twelve core values, including values relevant to patient care and values relevant to the lives of employed GPs. Values with high relevance were job satisfaction, the professional distance from patients, collaboration and collegial exchange, comprehensive care, adequate consultation time and availability to patients. Values with heterogeneous relevance were continuity of care, waiting times and medical autonomy. The value “availability” of employed GPs to patients was associated with both patient care and personal life. The limited availability of employed GPs was accompanied by tensions between these two trends and other values.

**Conclusion:**

The values of employed GPs are partly consistent with the current WONCA definition of general practice. There were also indications of new values. The increase in the proportion of employed GPs implies a need to reflect on the core values of general practice, taking into account factors on the part of employed GPs, patients, and practice organisation.

**Supplementary Information:**

The online version contains supplementary material available at 10.1186/s12875-023-02255-7.

## Background

The current definition of general practice by the World Organization of National Colleges, Academies and Academic Associations of General Practitioners/Family Physicians (WONCA) reflects the role of general practitioners (GPs) in Europe [1]. This definition includes established “core values” of general practice, such as holistic, comprehensive care or continuity of care, which are also part of the definition of core values of some European professional associations in General Practice [2]. Definitions of General Practice in Europe are developed within professional networks and are the subject of decision agreed at their general meetings.

Core values in this sense, describe a professional consensus on deeply held views of physicians that characterize the profession and guide their beliefs. They shape the profession, determine attitudes to professional responsibility and regulate actions in the professional context [2–4].

Therefore, consensus on the values of General Practice is documented. It may be based on a particular vision of General Practice, e.g. a model of a self-employed GP in a single practice, which does not fully correspond to the reality in several countries. For example, we observe a feminization of medicine, the choice of General Practice in anticipation of a good work-life-balance or family compatibility [5, 6] and the trend towards better self-care. The latter item was included in the Geneva Declaration of the World Medical Association in 2017 [7]. A change in the professional role and the core values of GPs is being discussed in several countries. This concerns, among other things, the balance between work and private life.

Directly care-related aspects are also increasingly discussed when talking about changes of the professional role [8, 9]: In 2016, Hashim abstracted the core values available in the literature worldwide to five principles of family medicine: compassion, generalism, relationship continuity, lifelong learning, and reflective mindfulness [4].

In several countries such as the Netherlands and Ukraine, coordination of care and collaboration among GPs have been identified as core values [10, 11]. The desire to work in groups indicates an increased importance of collaboration. Teamwork is also mentioned in the future positions of the German College of General Practitioners and Family Physicians [12]. Obviously, the number of discussed values and their aspects in General Practice is high.

In some European countries, the changing role of GPs has been accompanied by a trend towards practice-based employment: Austria allowed self-employed GPs to employ other GPs in 2019 [13]. In the Netherlands about 12% of the GPs were employed in 2012 [14].

In Germany, the majority of primary care physicians are self-employed. However, between 2006 and 2020, the proportion of employed physicians among GPs in Germany has increased from 3.1% to about 20% [15]. Employed GPs in Germany work directly for one or several self-employed GP(s) and have at least 5 years of training in General Practice training or internal medicine. This means, that employed GPs are paid directly and usually on a regular basis by an employing GP, who is remunerated according to the services provided in the practice. Parents and women in particular prefer the employment status [16]. Employed GPs work fewer hours than self-employed GPs [17].

These developments raise questions about continuity, access, and their impact on patients [18, 19] in an international context. Due to the shortage of GPs in several countries, high-quality care is partly dependent on employed GPs. Their value orientation is therefore of interest.

So far employed GPs do not seem to be explicitly considered in the discussion about the core values of GPs and the European definition of General Practice. To our knowledge there is no research on the value orientation of employed GPs regarding the provision of medical care. The aim of this study is therefore to qualitatively describe the values of employed GPs and the manifestation of these values in Germany. The research questions are:

1. What values do employed GPs have regarding their professional role?
2. To what extent are these values manifested in practice and what factors are associated with their manifestation?

The term “values” in this work refers both to core values already established in the definition of General Practice and to aspects of values currently under discussion or emerging from the data.

## Methods

To explore the values of employed GPs in a professional context we conducted 17 semi-structured telephone-interviews. Due to the lack of previous research on employed GPs we conducted an exploratory, qualitative study. Ethical approval was obtained from University of Heidelberg (S-986/2020). For reporting we followed the Consolidated Criteria for Reporting Qualitative Research Checklist (Additional file 1).

### Research team

The research was conducted by two master’s students in Health Services Research (LH, LB, both female), a part-time employed GP and researcher (SS, female, MD), a sociologist (CU, female, PhD), an implementation scientist (MW, male, Prof.) and a self-employed GP and researcher (FPK, male, Prof.) at the Department of General Practice and Health Services Research, University Hospital Heidelberg. All authors were experienced in qualitative research, have brought their background to the analysis, and reflected on the data from their own researcher’s perspective.

### Participant selection

Approximately 89% of the German population is covered by the statutory health insurance system, while the much smaller proportion is privately insured. All German GPs who are entitled to claim from the statutory health insurance are obligatory members of the Association of Statutory Health Insurance Physicians. The contact details of employed GPs can therefore be obtained directly from a publicly available search function. In our case we used the website of the Association of Statutory Health Insurance Physicians of Baden-Württemberg [20].

All employed GPs working in two neighbouring districts (city and county) in southern Germany were invited to participate in the study by SS, LH and LB to participate in the study by e-mail or fax on March 31, 2021, and were reminded by telephone from April 12, 2021, in case of non-response. The counties are not identified for privacy reasons. GPs with at least 5 years of training in General Practice or internal medicine, with a license to practice as GP and working for self-employed GPs in practices, were included. This information could also be obtained from the abovementioned website. Employed GPs working for employing GPs who are not entitled to claim from the statutory health insurance, could not be included. A purposive sampling strategy was used, focusing on a maximum variation of gender, age and practice type (single practice, joint practice, medical care center) to allow a broad representation of existing aspects.

Groups that were not adequately represented in the first round (male physicians and medical care centers) were subsequently invited by personalized letter, indicating the addition of a convenience sampling strategy. Participants provided written, informed and unsolicited consent. They were informed about the aim of the study, which was to learn about the daily life of employed GPs. There were no dropouts.

### Data collection

The interview guide (Additional file 2) was developed in an interprofessional team (CU, FPK, LB, LH, MW, SS) based on a literature review including definitions of General Practice by WONCA [1], and the German College of General Practitioners and Family Physicians [21]. It includes motivation for employment, personal experiences during employment, the relationship with employing physicians and the potential for improvement. In addition, self-reported sociodemographic data on the employed GPs and the practices were collected.

The interview guide was piloted and subsequently concretized with the help of three personal contacts who met (or until recently met) the inclusion criteria and subsequently concretised. The pilot interviews were not included in the further analysis.

The interviews were conducted by telephone between April 13, 2021 and May 26, 2021 by LH, LB and SS. They were audio recorded and a postscript was prepared. The interviews were transcribed. During the final interviews, no new topics were addressed by the participants. Further data collection was deemed redundant and data saturation was assumed. Furthermore, in a systematic review identifying studies that used empirical data or statistical modelling to assess saturation, 9–17 interviews were considered sufficient [22].

### Data analysis

We used a qualitative content analysis, which is appropriate for identifying relevant themes with an exploratory study. Because of its flexibility and practicality, we followed Kuckartz [23] procedure. The analysis of all initially collected qualitative data was performed by a junior researcher in the study team (LH): After familiarization with the material through repeated reading and case summaries (LH), the main categories were primarily formed and defined deductively based on the frameworks of European WONCA Definition of General Practice/Family Medicine [1], the definition of the Specialty General Practice by the German Society for General and Family Medicine [21], the World Health Organization’s global strategy on people-centered and integrated health services [24] and supplemented by other available literature (Additional file 3). The following categories were created within MAXQDA software: continuity, comprehensive care, collaboration and collegial exchange, waiting times, professional distance, work satisfaction and medical autonomy, job satisfaction, Availability and private life. Deviating from Kuckartz, subthemes were also generated inductively. The robustness of the data analysis was increased through researcher triangulation: Group discussions and reflections were conducted in the interprofessional study team with experienced qualitative researchers (SS, FPK) as well as in qualitative method workshops with junior and senior researchers (CU, MW), a summer school (LH), the 55th Congress of General Practice and Family Medicine (LH, SS) and in the Employment Working Group of the General Practitioners’ Association in Baden-Württemberg. The coding and overall analysis of the collected data were discussed and finalized with an experienced member of the study team (SS). After the complete coding of the material, the identified values were subdivided according to their relevance, taking into account the respondents’ subjective classification of each value in relation to the life of employed GPs, patients, the profession and other values. This resulted in values with “high and heterogeneous relevance”.

## Results

### Sample

We conducted 17 interviews, which lasted on average 50 min (minimum 22; maximum 83 min). The sociodemographic and work-related characteristics of the employed GPs interviewed are shown in Table 1.

**Table 1:** Sociodemographic and work-related characteristics of interviewed employed general practitioners (n = 17)

Characteristic		n	(%)
Family Status	Single Partnership Married	2 2 13	12 12 76
Gender	Female Male	14 3	82 18
Practice Type	Single Joint Medical care center	7 4 5	44 25 31
Age	37-44 45-52 53-60 n. a.	6 3 6 2	35 18 35 12
Experience in Family Practice^1^	1-5 6-10 11-15 16-20 n. a.	6 5 3 2 1	35 29 18 12 6
Working hours per week	16-25 26-35 36-45 41-50 n.a.	6 6 3 1 1	35 35 18 6 6
Children	∅	1.8	

^1^ years, full time equivalent

### Results: values of employed general practitioners

We found 12 values of employed GPs in their professional context. They differ in their relevance for the interviewees. First, the identified values are presented from the perspective of the employed GPs and structured according to the observed relevance. Then the implementation of the values (Table 2) and associated factors (Table 3) are reported from the perspective of the employed GPs. Citations originate from different interviewees.

**Table 2:** Manifestation of values with high relevance and associated factors from the perspective of employed general practitioners

Value	Manifestation
job satisfaction	Work satisfaction is enforced by abandoning bureaucratic work through task distribution in the practice, practicing different specializations and subjects (practicing the “*hobbyhorse*”). Family obligations are prioritized to actions that cause work satisfaction.
professional distance to patients	Mainly not realized because of intrinsic factors and factors learned in the hospital, like high sense of responsibility and availability during and outside working hours. Medical exchange with employing physicians helps with the distance.
Collaboration and collegial exchange	Exchange with employing physicians is broader than with other employed GPs, e.g. exchanging medical knowledge independent of current patients. More exchange in teams is needed instead of bilateral communication.
comprehensive care	Manifestation varies. Some say, they provide “everything“, e.g. diagnostics, prevention, and socio-legal communication. However, certain types of treatment, e.g. prevention against COVID-19, are only partially provided. The reasons for this are the preferences of the employed GPs, the lack of training, the time constraints of appointments for chronic patients and the lack of compatibility between their family and acute patients due to the difficulty of scheduling. In some cases, the range of services offered in the practice is limited by practice guidelines and time schedules, e.g. services not covered by the statutory health insurance and psychological problems.
availability of employed GPs to their patients versus private life	Compromises are made in the realization of value orientation in the tension between availability and family responsibilities: For example, inconvenient home visits are made but documentation is postponed. Patient needs are relevant: In most cases, work is not finished until the care of all patients is covered.
access: sufficient consultation time	Some employed GPs let patients talk and take time for phone calls and research. They conduct psychological and psychosomatic discussions with patients, because the profession’s self-image as a conversation-intensive discipline - even if not of sufficient length. Consultations in uncomplicated acute cases are kept short. Time conflicts arise during open consultations, when appointments and acute consultations overlap and during home visits. Employed GPs can partly influence individual appointments but can only change the planned appointment rhythm to a limited extent. Appointment scheduling by medical assistants is perceived as only partially accurate due to a lack of insight into the duration of treatment events. Deviations from the schedule are mostly accepted by the employed GPs if the request is perceived as important and cannot be postponed.

**Table 3:** Implementation of values with heterogeneous relevance and associated factors from the perspective of employed general practitioners

Value	Manifestation
continuity: coordination between providers	In most cases, detailed electronic documentation, coordination via practice software and contact with colleagues - even outside regular working hours – is sufficient. Verbal or phone handovers to ensure the flow of information are sometimes carried out but are not desired by all colleagues or occur only by chance. Coordination between providers is more likely to be successful if the providers know each other. However, duplicate anamnesis cannot always be avoided. Coordination is less successful when boundaries are crossed by the employing physicians, such as the changing medications without employed GPs knowledge and information sharing.
continuity: knowing the patients	Most employed GPs know their patients well, e.g. their names, working- and living-conditions and their relationships by asking the patients or other providers. Older patients with home-visits and/or social problems are known better than others: *‚I know my patients, I know them by name. […] maybe not for every patient, if somebody has the flu I don’t care, but if somebody has a special situation, or if somebody has been beaten, or if there is something to organize – or with child-rearing issues I really know about every step they take“.* The changing duration of the physician-patient-relationship seems to play a greater role in getting to know patients at the beginning of the relationship and becomes less important as the relationship continues. A holistic approach to patients, a high motivation of employed GPs, frequent consultations on a regular basis, having an own patient base and a sufficient consultation time can contribute to the knowledge about patients. The relevance of the availability of employed GPs (e.g. by phone or time in the practice) for patient knowledge is controversial.
continuity: duration of the physician-patient-relationship	It mostly depends on the willingness of the employed and employing physician to extend the employment contract.
continuity: care by a reference provider	Some employed GPs have their own patient base through disease management programs and health screenings. The reasons for this, despite low interest, are the desire not to burden colleagues and the emotional closeness to patients. Other employed GPs report a change in providers, because the process structures in their practices do not allow for care by reference physicians without increasing availability. Care by employing physicians without the consent of employed GPs and the increasing transfer of patients from employing physicians to employed GPs also contribute to care without reference physicians.
waiting times	Manifestation depends mostly on physician assistants and patients. Employed GPs try to reduce waiting times by working quickly and recommending doctors with higher availability to their patients. However, waiting times are unsatisfactory because office procedures leave no time for assessments, home visits, or requests, and the unselected patient population makes planning difficult. In two medical centers, rapid care for emergency patients is achieved by assigning doctors exclusively to acute consultations.
medical autonomy	Some employed GPs experience their work as self-sufficient, medically free, and independent. In some practices, employed GPs have the opportunity to make medical decisions regardless of financial consequences, as this is part of the practice culture. Others report that they have little influence, e.g. on patient selection. Some have implicit or explicit guidelines to consider profitability. However, some of them refuse to provide services if they do not consider them medically useful or if the consultation time is too short. For services not covered by the statutory health insurance, the “selling” of services does not correspond to the self-image of employed GPs.

### Values with high relevance

#### Job satisfaction

According to some employed GPs, job satisfaction leads to better patient care:

*“If the doctors are more satisfied, I think they can […] sometimes provide better medical care […]”*

and is therefore not only an issue for employed GPs themselves, but also for patients. Job satisfaction is also mentioned in the context of the type of work e.g. practicing different specializations and subjects and avoiding bureaucratic work.

#### Professional distance from patients

Employed GPs seek to maintain a professional distance from patients, mainly for reasons of self-care; patient benefits of professional distance are not mentioned within in the interviews. However, the absence of disadvantages of a professional distance for the patient is relevant for employed GPs.

#### Collaboration and collegial exchange

Employed GPs, employing physicians and patients benefit from teamwork and exchange between colleagues within the practice and this is seen as valuable:

*“What we do a lot […]: Please come here and take a look at this rash, or: Can you help me? […] everything that´s collegial is very valuable to me, so it’s more valuable to me than the things that bother me, because I also believe that our patients benefit immensely from it.”*

#### Comprehensive care

Comprehensive care by individual providers is usually considered necessary. The inability to offer meaningful services and existing restrictions on treatment occasions, e.g. exclusive acute consultations of individual providers, are criticized. Comprehensive care by the entire practice is also relevant for the employed GPs, and the necessary range of services is described as “*from athlete’s foot to brain tumours”*. Multiple views of diseases, specialization and increased specialist expertise can be achieved, and diagnostic possibilities can be used when the entire practice works comprehensively.

#### Access: sufficient consultation time

When it comes to access, employed GPs see sufficient consultation time as highly relevant. It is described as part of the employed GPs’ philosophy or professional role:

*“GP just means […] I have many patients with psychosomatic or psychological problems that you just can’t deal with in five minutes”.*

Employed GPs would like to have more time window per consultation as a long consultation makes medical sense. The time is needed to curb polymedication, treat chronic and mental illness, and translate diagnoses into therapies. They would like to see fewer patients “pushed in” so that their work is more valued.

#### Availability of employed GPs for their patients versus employed GPs private lives

By availability, we mean being available for patients or care-related tasks. Availability has many consequences for employed GPs (e.g. less free time, family conflicts and difficulty in maintaining emotional distance from patients) and for patients (e.g. shorter waiting times, longer consultations and longer office hours of the practice). It is therefore important for the realization of other values, e.g. consultation time, waiting time, the care by a reference provider and private life. By reference provider we mean a general practitioner, who coordinates care, feels responsible for the patient, has the patient on his or her caseload, or to whom the patient feels connected. Limited availability can lead to tensions between values and the need to choose between them. Possible factors for the availability are the day of the week, the location of care, the mission, and policies of the practice, the reason for and urgency of the consultation, the number of colleagues and the existence of a reference provider. Availability functions as a link between private and directly care-related values, as it determines the feasibility of directly care-related values but has an opposite effect on the feasibility of private life. This means, that private life values can become indirectly relevant to patient care through the link of availability: The involvement of employed GPs in private life may affect the provision of adequate consultation time, waiting time or care by reference provider.

Some of the employed GPs consider their own availability less important than that of employing doctors, who also have limited availability. Most employed GPs are more concerned with family obligations than with the consequences of low availability for patients. For example, the dissatisfaction of patients due to low availability is accepted by employed GPs, because the desire to be with the family predominates. Another example is the illness of children of employed GPs, in which normatively the family value prevails over availability:

*“…it was also very difficult to then hand over the sick child to someone else […] where it was then simply clear to me that it was not worth it to always torture myself [to the practice].”*.

### Values with heterogeneous relevance

#### Continuity

While coordination between providers and knowing the patient are important parts of continuity, the duration of the doctor-patient-relationship is only partially relevant and the care by a reference provider is perceived as primarily irrelevant. Coordination between providers is particularly important for employed GPs because the care of a patient is often provided by different physicians. It contributes to the knowledge about diagnoses:

*‘For that you need time and leisure to really do handovers, […] The patient did not have a follow-up appointment […] and then came back to me at some point and I looked in this lab and hepatitis C was positive. […] where I think that at some points you have to make sure that you´re doing handovers and that things don’t slip up.”*

Knowing the patients is seen as a resource for a needs-based-treatment and can reduce the time needed for consultation. It is also a factor in employed GPs’ job satisfaction. It is less relevant in a short-term employment with priority treatment of patients with acute causes. The heterogeneity of the relevance of the duration of doctor-patient relationship is due to the growing knowledge about patients (especially in the first phase of the relationship) and the desire not to be tied to a practice. Care by a reference provider is mostly irrelevant for employed GPs, as coordination between providers usually works well. More important are free time, job satisfaction, professional distance and, in some cases, short waiting times. The resulting plurality of providers and their different opinions and specializations are seen as beneficial for care and for the physicians themselves. Exceptions are made for home visits, preventive care, rehabilitation, and changing providers within the same visit.

#### Waiting times

Short waiting times are particularly important for the interpretation of diagnostic consultations. When choosing between waiting times and care by a reference provider, employed employed GPs tend to focus on low waiting times, as some suggest reducing them by foregoing care by a reference provider. Round-the-clock care is also sometimes seen as more important than care by reference providers:

*“We try, if it is somehow possible, not to let the patients fixate so much on the individual physician, […], because we try to ensure this round-the-year and round-the-clock care.”*

Short waiting times are less important for preventive care and when compared to waiting times for specialists:

*“If you want to have a check-up with a defined GP, you have to wait four weeks for the appointment. I think that’s really short compared to specialists.”*.

#### Medical autonomy

Medical autonomy is most often seen in a conflict with economic pressures. Medical autonomy is important to employed GPs, when they compare it to other providers or working environments (e.g. inpatient sector) - especially for newcomers - and when employing doctors do not follow their own guidelines or intervene in medical affairs without being asked:

*“he says […] cold medicines all go on a green prescription [paid by the patient] and I then represent that to my patients. Then they trot over to the boss, cajole him and then it’s given on a red prescription [paid by the statutory health insurance] and of course that annoyed me massively”.*

Restrictions of Medical autonomy are better accepted when employed GPs understand the reasons for the restrictions. It is also better accepted by employed GPs when discussions with the employing physicians are seen as learning opportunities, when exceptions can be made for special circumstances and when their working philosophy is similar to that of the employing physicians’. This similarity may result from the adaptation of their working methods over time:

*“In the beginning, there was much more authority, but now I work quite autonomously. After twenty years, of course, you also adapt very much to each other, so there are not so many conflicts in the sense that he has to dictate certain things to me, because we are very similar.”*.

The manifestation and associated factors from the perspective of employed general practitioners are presented in Tables 2 and 3. All statements are derived from the responses of the interviewees (n = 17). A comparison of Table 2 and the relevance of availability and private life reveals a discrepancy between intrinsic value orientation and implementation, e.g. when it comes to the professional distance to patients. In particular, the value orientation of availability and the private life (high relevance of family time) differs from the practical implementation, as compromises and consideration of the patient needs are taken into account.

## Discussion

Important values for employed GPs in the professional context are job satisfaction, a professional distance from patients, cooperation and exchange with other providers, comprehensive care, sufficient consultation time, availability and family. Continuity, waiting times and medical autonomy are only partly perceived as important (interpersonal and intrapersonal) or less important than other values. Tensions occur between values, especially due to the limited availability of employed GPs for patients. Manifestation factors can be at the practice, patient, and physician levels.

The overall view of important values of employed GPs may give the impression that there is little focus on patient-centeredness, with values such as job satisfaction, professional distance from the patients and collaboration and collegial exchange being mentioned. However, from the perspective of employed GPs, these values are also relevant to the patients and lead to better medical care or at least do not result in disadvantages for the patients.

In our study, employed GPs perceive a tension between private life and other values to due to their limited availability. Availability is thus a central factor in the compatibility of values directly relevant to care but has an opposite effect on compatibility with the private life. The importance of availability may give the impression that employed GPs with low availability cannot provide continuity, adequate consultation time and rapid access: Employed GPs must prioritize values. However, low availability does not necessarily lead to poorer care from the perspective of employed GPs perspective: Reference providers are seen as having little relevance to care when good coordination between providers exists. Similarly, Bodenheimer et al. [19] and Pannatoni et al. [18] show that better patient satisfaction with part-time primary care physicians does not depend solely on waiting times and care provided by referral physicians; a trusting therapeutic relationship can also be established despite relatively low availability (to some extent). Employed GPs need support in making complex value judgments and in providing adequate continuity. Bodenheimer gives some examples [19]. In our study, availability can be influenced, e.g. by good working conditions, leading to less absenteeism. It should not be ignored that GPs increasingly demand a private life, as the realization of a private life is indirectly relevant to patient care. The choice of employment with the motivation to reconcile family and work may suggest a better compatibility than self-employment. However, according to the present results, it can by no means be assumed that this compatibility in employment is comprehensive enough for the employed GPs.

Knowing the patients is seen as particularly important for the continuity of the relationship in this study. Care provided by reference providers tends to be perceived as less important, the latter especially when coordination between providers works well. The high relevance of patient knowledge combined with the low relevance of care by referring physicians may be seen as a contradiction, but patient knowledge is built up not only through the quantity but also the intensity and quality of consultations. However, a systematic review has shown an association between less care from reference physicians and patient mortality [25], and the presence of a reference physician reduces hospitalization and use of out-of-hours care [26]. Future research could consider organizational aspects such as the quality of coordination between providers could be considered.

The theme of professional distance to patients was also found in a multicentric qualitative study by Le Floch et al. GPs wanted to “control the level of involvement with their patients” and described an “ability to balance empathy with professional distance” [27]. Employed GPs in our study seem to focus on self-care in the area of tension between distance from the patient and the relationship with the patient. They also find it more difficult to balance professional distance with the patient relationship, describing intrinsic factors and a high sense of responsibility as reasons for this challenge.

Regarding medical autonomy, hierarchies exist in practices due to the different roles of self-employed and employed physicians despite equal professional qualifications. The realization of physician autonomy is described as heterogeneous in the present study. A qualitative study from the United Kingdom shows a negative perception of realization by employed primary care providers, namely disempowering and disadvantageous hierarchies [28]. The values of employed GPs found in this study overlap with the professional definitions of WONCA (e.g. comprehensive care, continuity and access), as well as with values currently discussed in the literature (private life, job satisfaction, collaboration and exchange). Thus, the values of employed GPs are partly consistent with the discussion about the professional profile and the definitions of General Practice. They show that employed GPs are partly oriented towards established values [1].

Our study also found values that were not previously included in the discussion of the changing professional role and in the GPs definitions: Professional distance from patients, availability for patients and medical autonomy of the physicians.

### Strengths and limitations

Strengths of the exploratory, hypothesis-generating study stem from the data collection and analysis: We achieved a wide variance in the sample in terms of practice type and age of the employed GPs. The comprehensibility of the interview questions was increased by piloting of the interview guide. The length of the interviews (Ø 50 min) allows for in-depth insights. The deductive-inductive approach helped to identify central values. The entire research process was complemented by the extensive exchange in the interdisciplinary research team.

Limitations arise from the regional focus of the study. Even if urban and rural practices were integrated, other regions may have different care structures, leading to different results. Personal relationships between one interviewee and SS as a recruiter may have increased social desirability. To mitigate this risk, this interview was not conducted by SS. Voluntary participation introduces a selection bias; it is possible that particularly motivated or distressed employed GPs participated. Recruitment of a broad gender variation was only partially successful (3 of 17 interviewees are male), as only 30% of employed GPs working in practices with approval for claiming from statutory health insurance in Germany are male [29]. The centrality of the value availability may have arisen due to the high proportion of part-time workers in this study, given the high percentage of employed GPs working less than 30 h a week in Germany (45% among employed GPs working in practices with statutory health insurance participation (2020) [29]). For data protection reasons, pseudonymised information on the employed GPs cited cannot be given. The frameworks, that were used in clinical practice, were not developed for qualitative research. We are not aware of frameworks for core values in General Practice, that were developed for research. Due to the exploratory and qualitative character, no generalizations can be made. However, the study design is considered appropriate for the hypothesis-generating approach. Representative quantitative surveys in the group of employed GPs could follow to verify the results.

## Conclusion

Several values are important for employed GPs, with availability to patients being crucial. It serves as a link between private life and patient care and involves several areas of tension. Trade-offs in the realization of values are often multifactorial, with factors related to practice organization, physicians, and patients. The values of employed GPs are partly consistent with the professional definition of General Practice and the discussion about the professional profile. The increase in the number of employed GPs implies the need to reflect on the core values of General Practice and to consider employed GPs in the promotion of work-family balance. The extent to which established and new values play a role for the general practice profession remains open and can be explored in further research projects and professional policy discussions. The future will show to what degree employed GPs, employing GPs and patients need to adapt to both, changing health care systems and changing professional values in General Practice.

### Electronic supplementary material

Below is the link to the electronic supplementary material.

Additional File 1

Additional File 2

Additional File 3

## Data Availability

The datasets generated and analyzed during the current study are not publicly available due to European provisions for data protection, of which the interviewees were assured, but are available from the corresponding author on reasonable request.

## Abbreviations

GP: General practitioner
WONCA: World Organization of National Colleges, Academies and Academic Associations of General Practitioners/Family Physicians
