# Supplementary material for: Core values of employed general practitioners in Germany – a qualitative study
Source: BMC Prim Care. 2024 Jan 6;25:14. doi: 10.1186/s12875-023-02255-7 (PMC10770961; doi:10.1186/s12875-023-02255-7)
Supplement: Supplementary file 2 — Additional File 2 [file 12875_2023_2255_MOESM2_ESM.docx]

| 1. **Introduction: „How did you get into this?“ (Reasons/Motivation)** |
| --- |
| **Tell us: How did you come to work as an employee?**   - What considerations influenced your decision? (Money, free time, children...) - What were the reasons for your decision? - Why did you not open up your own practice? |
| 1. **Topic: „What is it like?“ (personal experience)** |
| **What is a typical working day like for you as an employed GP?**   - What are your tasks? - How are your working hours organised? - How do you handle overtime? - How are appointments organised in your practice? - What is the role of limited working hours in the organisation? What is the impact on the patients? - If practice participates in GP-centred care: What is the role of employed GPs play in GP-centred care? [GP-centred vs practice-centred? Enrolment practice (even if not a contracting partner?)   **How is your employment perceived by those around you?** [colleagues in general, practice team, private]   - What attitudes and reactions do you encounter? - How do you deal with them? - Do you encounter prejudice?   **What does your employment mean for the patients in your practice?**   - How does it affect patient care? - How is continuity of care organised? - How well do you know your patients? Do you see a difference to your employer? |
| 1. **Topic: Relationship: Employing GP – employed GP** |
| **How do the tasks of the employing GP differ from your own?**   - How is the distribution of tasks decided? How satisfied are you with this? - What role did you play in changing practice procedures during the COVID 19 pandemic?   **What information do you exchange with your employing GP?**   - How are decisions made? Are you satisfied with them? (e.g. holiday planning) - How do you deal with conflicts? (e.g. dissatisfaction with practice organisation). - To what extent are authority and directives an issue?   **What is the role of finance and remuneration in your practice?**   - How was your salary determined? What issues arose in the process? How satisfied are you with it? - What is the role of income/turnover in your practice? - How has the COVID-19 pandemic affected your income? (Short-time work, lay-off...)   **What role do legal agreements play between you?** [Employment contract, recourse, liability cases]   - What was discussed when the contract within the contracting process? - What is the role in everyday life? |
| 1. **Topic: Possibilities for improvement in the field of employment** |
| - What do you think needs to be changed in the working conditions regarding employment?   - If necessary, ask: In relation to your practice? If you imagine being employed forever? - Where do you see potential for improving patient care in terms of employment? |
| 1. **Topic: How will it develop?** |
| - What would you recommend to a colleague who is faced with the choice of employment or own practice? - How are you currently doing in your employment? - To what extent are you considering settling down? |
| 1. **Ending** |
| - We have now reached the end of the interview. Is there anything else you would like to add, anything that we have not yet discussed or not sufficiently discussed about employment? |
