# Supplementary material for: Core values of employed general practitioners in Germany – a qualitative study
Source: BMC Prim Care. 2024 Jan 6;25:14. doi: 10.1186/s12875-023-02255-7 (PMC10770961; doi:10.1186/s12875-023-02255-7)
Supplement: Supplementary file 3 — Additional File 3 [file 12875_2023_2255_MOESM3_ESM.docx]

**Definition of Categories**

**Comprehensive care**

Aspects that address the scope of care, such as health promotion, preventive care, curative care, rehabilitation, acute and chronic care, supportive care, physical, psychological, and social perspectives, clinical, ethical, and humanistic aspects of the physician-patient relationship, broad responsibility of an unselected patient population, and range of services. The category answers the question: ‘What is provided (and what is not)?’. Comprehensive care can include comprehensive care by a physician (the scope of care provided by individual providers) and comprehensive care by a practice (aspects relating to the scope of care provided in practices with employed physicians).

The distinction with continuity is as follows: Comprehensive care describes the extent to which a physician provides all services, but not always in relation to one specific patient. Disease-independent continuity describes whether a patient is cared for by one provider for all diseases.

Sources of category and definition: [1,2]

**Continuity**

The extent to which a series of discrete health care events are experienced by patients as coherent and connected over time, and consistent with their health needs and preferences [3].

*Coordination between providers*

The term is also known as 'management continuity' and a part of 'integrated care'. It refers to the degree to which health care for specific users is coordinated between providers and/or institutions. The category includes communication between providers (=informational continuity), e.g. documentation and cooperation (=teamwork or joint care planning). This category does not address whether different providers are involved, but how care is arranged between providers if there are several. This category does not include exchanges between providers, that occur independently of a division in care processes (🡪 voluntary exchanges between colleagues).

Sources of category and definition: [1, 4, 5-8].

*Relationship continuity*

This category describes the continuity of care in relation to a specific providers. It is distinguished from coordination between providers as follows: This category addresses **whether** multiple providers are involved, it does not address **how** care is provided when multiple providers are involved.

Sources Category and Definition: [1,2,5-8]

*Duration of the physician-patient relationship*

Duration of the physician-patient relationship with a particular physician. Refers to longer periods of time and not the occasional absence of physicians.

*Knowing patients*

Find out about and remember characteristics of patients, be able to assess patients. Have knowledge of patient behavior. Know biographical and medical information.

*Have reference* providers

This category includes statements that provide information about whether and in what context patients are assigned to a particular providers or feel they belong to them. It also includes the extent to which several providers provide care or whether patients have quantitatively few primary care providers (longitudinal continuity). Statements on the extent to which patients have primary providers for home visits are also included. This category also describes the extent to which patients feel they belong to certain providers, regardless of their illness or depending on certain illnesses. This aspect can be distinguished from the ‘comprehensive care’ category as follows: Continuity describes whether patients are treated by a reference provider for all diseases. Comprehensive care describes the extent to which a providers offers all services, but not always in relation to one patient.

**Collaboration and intercollegiate exchange**

Means collaboration that is independent of the division of the care process, but supportive exchange with other providers.

**Waiting Time**

Refers to time that patients have to wait to receive care: Both long-term waiting times for appointments, feedback, diagnostic results, and other components of care, and on-site waiting times at the practice. The category also refers to practice opening times (e.g.,

nights, weekends), as these affect the waiting time.

**Medical autonomy**

Aspects relating to freedom in therapeutic decisions and therapeutic options, e.g., restrictions and increased freedom.

**Professional distance**

Aspects that express the emotional closeness or distance to the patients and to care, e.g. in distinction to other areas of life such as the private life. This includes the influence of patients and work at private life, the experience of intimate situations, empathy, the self-openness of physicians towards patients, and interdependence, e.g. in the sense of influence on the private activities of physicians. Closeness can be a characteristic of the relationship as well as a temporary state.

**Job satisfaction**

Concerns the job satisfaction in relation to patient care and the perceived enjoyment of patient care.

**Private life**

The role of leisure time, family, and other private commitments in patient care. Family includes both biological kinship and family in the sociological sense: partnership, marriage, child-rearing, and similar arrangements.

**Availability**

Means the time availability of providers for patients. It includes availability outside of working hours, the occurrence of overtime, availability in the practice and in the patient's home environment on a daily basis and in special situations, e.g. when there is a high workload in the practice. It also includes work in the home environment of the providers, e.g. via phone or messenger app and work that does not exclusively require direct contact with other providers or patients, but which is of direct benefit to patient care.

**References**

[1] The European Definition of General Practice / Family Medicine [Internet]. European Academy of Teachers in General Practice (Network within WONCA Europe). 2011 [last modified 2011; 10.08.2021]. Available at: https://www.woncaeurope.org/file/3b13bee8-5891-455e-a4cb-a670d7bfdca2/Definition%20EURACTshort%20version%20revised%202011 .pdf

[2] Definition of General Practice [Internet]. German Society for General Practice and Family Medicine. 2002 [last modified 2002; 10.09.2021]. Availabe at: https://www.degam.de/fachdefinition.html

[3] WHO global strategy on people-centred and integrated health services -Interim Report [Internet]. World Health Organization. 2015 [last modified 2015; 08.09.2021]. Availabe at: https://apps.who.int/iris/handle/10665/155002

[4] Berger J, Distler L, Kloster A, Marg A, Rösel T, Weiler J et al. Modellprojekt sektorenübergreifende Versorgung in Baden-Württemberg - Projektbericht. Ministerium für Soziales und Integration Baden-Württemberg. [Model project intersectoral Care in Baden-Württemberg – Project Report. Ministry for Social Affairs and Integration. 2018 [last modified 2018; 08.09.2021]. Availabe at: https://www.gesundheitsdialog-bw.de/fileadmin/media/Modellprojekt_SueV/SueV_BW_Abschlussbericht.pdf

[5] Uijen AA, Schers H, Schellevis FG, van den Bosch WJHM. How unique is continuity of care? A review of continuity and related concepts. Fam Pract. 2012: 29:264–271.

[6] Meiqari L, Al-Oudat T, Essink D, Scheele F, Wright P. How have researchers defined and used the concept of ‘continuity of care’ for chronic conditions in the context of resource-constrained settings? A scoping review of existing literature and a proposed conceptual framework. Health Res Policy Syst. 2019: 17:27.

[7] Freeman G, Hughes J. Continuity of care and the patient experience [Internet]. An Inquiry into the Quality of General Practice in England. The King’s Fund. 2010 [last modified 2010; 08.09.2021]. Availabe at: https://www.kingsfund.org.uk/sites/default /files/field/field_document/continuity-care-patient-experience-gp-inquiry-research-paper-mar11.pdf

[8] Salisbury C, Sampson F, Ridd M, Montgomery AA. How should continuity of care in primary health care be assessed? Br J Gen Pract. 2009: 59(561):e134-e141.
